# Stable hippocampal correlates of high episodic memory function across adulthood

**DOI:** 10.1038/s41598-025-92278-0

**Published:** 2025-03-14

**Authors:** Anders M. Fjell, Markus H. Sneve, Inge K. Amlien, Håkon Grydeland, Athanasia M. Mowinckel, Didac Vidal-Piñeiro, Øystein Sørensen, Kristine B. Walhovd

**Affiliations:** 1 Center for Lifespan Changes in Brain and Cognition, Department of Psychology, University of Oslo, 0373 Oslo, Norway; 2 Center for Computational Radiology and Artificial Intelligence, Department of Radiology and Nuclear Medicine, Oslo University Hospital, 0424 Oslo, Norway

**Keywords:** Aging, Episodic memory, Hippocampus, Magnetic resonance imaging, Brain activity, Atrophy, Neuroscience, Psychology

## Abstract

Some older adults show high episodic memory performance compared to same-age peers. It is not known whether their high function is caused by special brain features in aging, or whether superior memory has the same brain foundation throughout adult life. To address this, we measured hippocampal volume and atrophy, microstructural integrity by diffusion tensor imaging, and activity during an episodic memory encoding and retrieval task in cognitively healthy adults (*n* = 277, age 20.1–81.5 years). Atrophy was quantified by repeated MRIs (2–7 examinations, mean max follow-up time 9.3 years). Superior memory was associated with higher retrieval activity in the anterior hippocampus and less hippocampal atrophy. There were no significant age-interactions, suggesting stable correlates of superior memory function. Age-memory performance curves across the full age-range were similar for participants with high memory performance compared to those with normal and low performance. These trajectories were based on cross-sectional data but did not indicate preserved memory among the superior functioning older adults. In conclusion, the results confirm that aspects of hippocampal structure and function are related to superior memory, without evidence to suggest that the best performing older adults are characterized by special hippocampal features compared to their younger counterparts.

## Introduction

Some older adults show superior episodic memory function compared to same-age peers, and even perform on par with much younger persons^1,2^. The aim of the present study was to explore hippocampal contributions to high memory function in aging by investigating volume, rates of atrophy, microstructural integrity, and encoding- and retrieval related activity, which may all play a role in episodic memory in aging^3^. Although it is well established that different hippocampal features are related to episodic memory function, it is not known whether the contributions of these features to high episodic memory function are similar across the adult age-range. Several theories about episodic memory function in aging suggest specific hippocampal-dependent mechanisms, but it is unclear whether such hippocampus-memory relationships emerge in aging or can be seen also in younger adults. In particular, we aimed to test whether there are exceptional features of the hippocampus in aging that can help explain why some older adults perform at a very high level, or whether the neural correlates of superior memory are similar across adult life.

A yet unanswered question is whether superior episodic memory performance in aging is due to resilience to expected age-related brain decline^4^, or rather reflects life-long high function. The latter explanation is in line with research showing high correlations between cognitive function in early and later life, that individual differences in brain and cognition appear very stable over the adult lifespan, and that differences in change rate appear to be a modest source of individual differences among older adults^5–7^. Successful aging may thus have a foundation in early life cognitive function which then is continuously shaped in response to changing environments^3,5,8^. However, some studies have also found high-functioning older adults to show less memory decline over time^9–11^, although with some variation^12^. Hence, superior memory performance in older age may represent a mix of life-long high function and relatively less decline.

Few longitudinal brain imaging studies have addressed the topic of superior memory function directly, but cross-sectional studies have reported larger volumes of hippocampal and other brain structures when older high-performers are compared to their normal performing age-peers^2,9,12,13^. Such findings may reflect stable individual differences and do not necessarily imply brain maintenance^9,12^, a question which requires longitudinal data to address. Less whole-brain cortical volume loss in high-performers was reported in one longitudinal study^14^, and memory decline across the life-span is related to macro- and microstructural brain changes, especially of the hippocampus^15,16^. Hippocampal volume loss typically accelerates in older adults^17,18^, even in participants with very low risk of Alzheimer’s Disease (AD)^18^, with a lifespan trajectory mimicking that seen for episodic memory. Similarly, integrity of the hippocampus, as measured by diffusion tensor imaging (DTI), is reduced across the adult lifespan^15^. These hippocampal changes are not only benign, with more atrophy and integrity reductions being related to steeper decline of episodic memory, although the associations are usually modest^19–21^.

Measures of brain activity during episodic memory tasks could explain part of the remaining variance in memory performance not accounted for by hippocampal structure and atrophy. Maintained memory function has been related to preservation of functional memory networks including the hippocampus^22–24^, although others have argued that compensatory brain activity can support memory function in aging^25^. Hippocampal activity measured during episodic memory tasks may thus contribute to explain superior memory performance in some older adults. It is beyond the scope of the current manuscript to provide a full review of previous research in the field. For a more comprehensive review of functional brain imaging of episodic memory decline in ageing, see^26^, and for a review of successful memory aging, see^3^.

In the present study we perform a comprehensive, multi-modal investigation of hippocampal contributions to individual differences in episodic memory function, combining longitudinal measures of atrophy with volumetric analyses, microstructural analyses by use of DTI, and functional analyses by use of fMRI during episodic memory encoding and retrieval. The main question investigated was whether memory-hippocampus relationships emerged in aging or rather was age-independent, indicating stable associations across adult life.

## Materials and methods

### Sample

The participants were recruited from ongoing studies coordinated by the Center for Lifespan Changes in Brain and Cognition (LCBC) at the Department of Psychology, University of Oslo, Norway. The final sample consisted of all available participants February 5th, 2024 who were 20 years or older, with valid cross-sectional task-fMRI recordings during an associative encoding and retrieval task, diffusion tensor imaging (DTI) scans, and longitudinal T1-weighted MRIs for quantification of atrophy. The final sample consisted of 277 well-screened cognitively healthy participants (137 females, age 20.1–81.5 years at baseline, mean 49.2 years, SD = 17.7 years). 113 had two separate scanning sessions, 63 had 3, 65 had 4, 23 had 5, 7 had 6, and 6 had 7. The mean follow-up interval from baseline to the last scan was 9.3 years (2.5–17.3 years, SD = 4.2). The participants were compensated for their participation.

All participants gave written informed consent, and the Regional Ethical Committee of South Norway approved the study, and all methods were performed in accordance with the relevant guidelines and regulations. At baseline, the participants reported no history of neurological or psychiatric disorders or conditions, including depression, chronic illness, premature birth, learning disabilities, or use of medicines known to affect nervous system functioning. They were further required to speak fluent Norwegian and have normal or corrected-to-normal hearing and vision, and were required to score ≥ 25 on the Mini Mental State Examination^27^. Mean MMSE-score was 29.2 (*n* = 255, range 25–30, sd = 0.9, correlation with age *r* = − .12, *p* = .05). Participants were tested on Vocabulary and Matrix Reasoning subtests of Wechsler’s Abbreviated Scale Intelligence Scale (WASI) (Wechsler, 1999), and all scored within the normal IQ range (> 85). Mean IQ-score was 116 (*n* = 266, range 89–141, sd = 11.2, correlation with age *r* = .28 (*p* < .001). All participants remained cognitively normal throughout all follow-up examinations as evidenced by a neuropsychological exam. Participants were further excluded due to experimental and operator errors, low number of trials available for fMRI analysis (*n* = 24 from the total participant pool; <6 trials per condition of interest) and extreme head movement (*n* = 1; >1.5 mm mean movement), reducing the final sample to the above mentioned 277 participants. The sample partially overlaps with the samples used in Sneve et al.^28^, Vidal-Piñero et al.^29^, and^30^, which addressed different research questions, and did not report analyses targeting the question of age-specific relationships between memory performance and multi-modal hippocampal features. Further^15,30^, included participants from 6 to 4 years of age, respectively, focusing on lifespan development, in contrast to the present study on adulthood and aging.

### Experimental design—fMRI tasks

Participants were scanned using BOLD fMRI during an associative encoding task and an unannounced subsequent memory test after ≈ 90 min. After the encoding, participants were taken out of the scanner, and not given any specific instructions about what to do during the retention interval. Importantly, they were not informed that they would be tested for the encoded material. The fMRI task is described in detail elsewhere^28^. Briefly, the encoding and the retrieval tasks consisted of two and four runs, respectively, of 50 trials each. All runs started and ended with an 11s baseline period, which was also presented once in the middle of each run. The stimulus material consisted of 300 black and white line drawings depicting everyday objects and items. During encoding, the participants went through 100 trials and performed simple evaluations. A trial had the following structure: a female voice asked either “Can you eat it?” or “Can you lift it?” Both questions were asked equally often and were pseudorandomly mixed across the different objects. One second after question onset, a black and white line drawing of an object was presented on the screen along with response indicators, and participants were instructed to produce yes/no-responses. Button response was counterbalanced across participants. There were no correct or incorrect responses. The object remained on the screen for 2 s, when it was replaced by a central fixation cross that remained throughout the intertrial interval (ITI; 1–7 s exponential distribution over four discrete intervals).

During the surprise memory test, 200 line drawings of objects were presented, of which an equal number was old or new. A test trial started with the pseudorandom presentation of an object and the auditory presented question (Question 1) “Have you seen this item before?”. Each object stayed on the screen for 2 s, and participants were instructed to respond “Yes” or “No”. If the participant responded “Yes”, a new question followed (Question 2): “Can you remember what you were asked to do with the item?” A “Yes”-response to this question led to a final two-alternative forced choice question (Question 3): “Were you asked to eat it or lift it?” Here, the participant indicated either “Eat” or “Lift”. The contrast of interest consisted of recollection – miss memory conditions, computed for each participant, i.e. correct responses to all three questions vs. “No” or a wrong response to question 1. This contrast was chosen to maximize signal differences between conditions. For activity differences between various possible contrasts elicited by this paradigm, we refer to a previous publication.

The design efficiency was tentatively optimized to ensure sufficient complexity in the recorded time series (http://surfer.nmr.mgh.harvard.edu/optseq/).

### Analysis of behavioral data

Multiple behavior variables were extracted and used in the analyses. Number of correct recognized items (regardless of source), correct rejections, recognition misses, false alarms, and recollections were entered into a principal component analysis to extract a main component of memory performance which was used as the memory measure of interest in all analyses. This component explained 53.9% of the variance, with recollection responses showing the highest loading (0.96).

### MRI scanning

At baseline, imaging was performed at a Siemens Skyra 3T MRI with a 20-channel head-neck coil at Oslo University Hospital, Rikshospitalet. For functional imaging the parameters were equivalent across all runs: 43 slices (transversal, no gap) were measured using T2* weighted BOLD EPI (TR = 2390ms; TE = 30ms; flip angle = 90°; voxel size = 3 × 3 × 3 mm; FOV = 224 × 224; interleaved acquisition; GRAPPA = 2). Each encoding run produced 131 volumes while the number of volumes per retrieval run was dependent on participants’ responses (mean 207 volumes). Three dummy volumes were collected at the start of each fMRI run to avoid T1 saturation effects in the analyzed data. Additionally, a standard double-echo gradient-echo field map sequence was acquired for distortion correction of the EPI images. Visual stimuli were presented in the scanner environment with a 32-inch InroomViewing Device monitor while participants responded using the ResponseGrip device (both NordicNeuroLab, Norway). Auditory stimuli were presented to the participants’ headphones through the scanner intercom.

At the time of the fMRI scanning, anatomical T1-weighted MPRAGE images were also acquired, consisting of 176 sagittally oriented slices were obtained using a turbo field echo pulse sequence (TR = 2300 msec, TE = 2.98 msec, flip angle = 8°, voxel size = 1 × 1 × 1 mm, FOV = 256 × 256 mm). For DTI, a single-shot twice-refocused spin-echo echo planar imaging (EPI) with 64 directions: TR = 9300 ms, TE = 87 ms, b-value = 1000 s/mm^2^, voxel size = 2.0 × 2.0 × 2.0 mm, slice spacing = 2.6 mm, FOV = 256, matrix size = 128 × 130 × 70, 1 non-diffusion-weighted (b = 0) image. Another non-diffusion-weighted (b = 0) image was acquired with the reverse phase encoding for distortion correction.

**Fig. 1:**
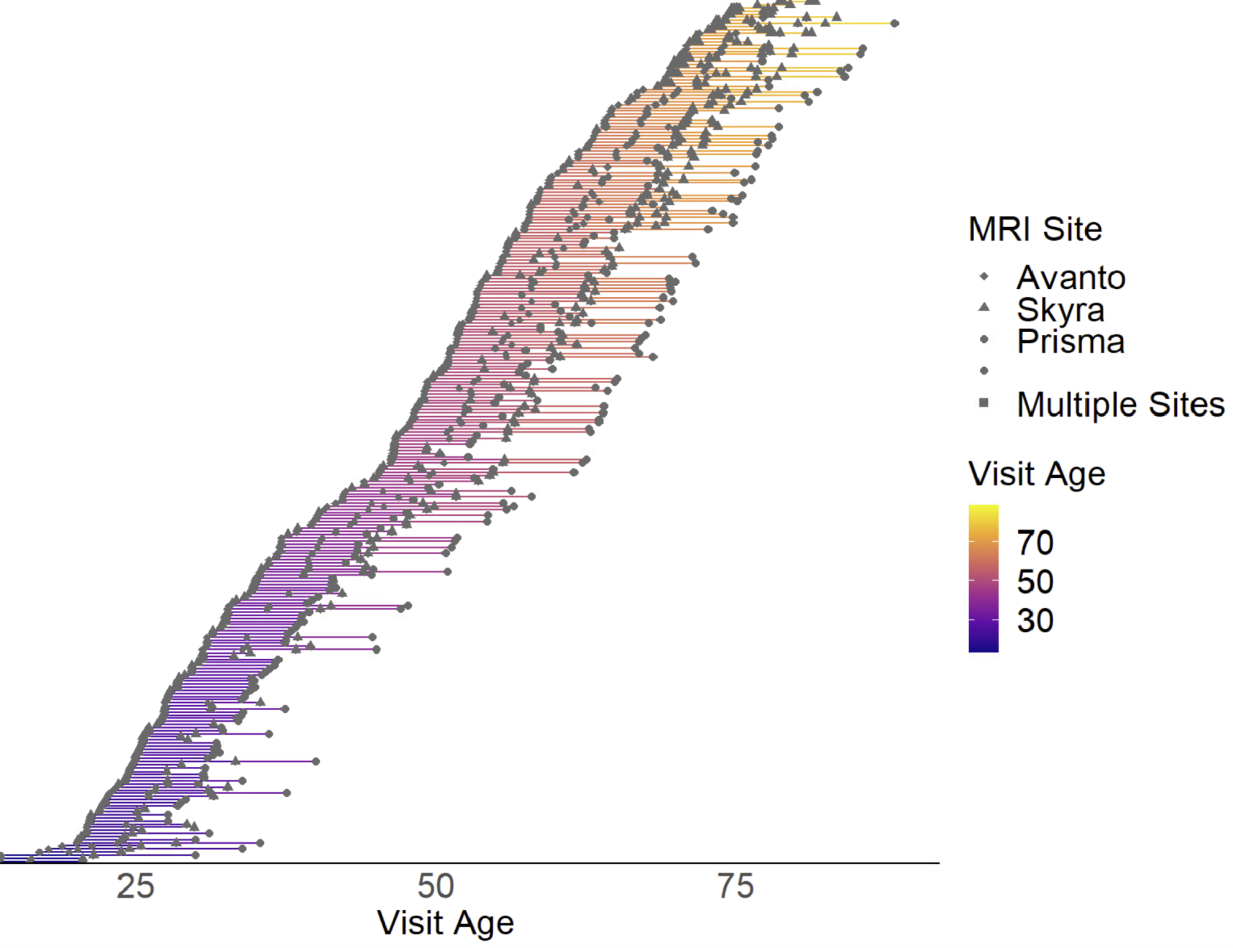
Overview of longitudinal data. Each line represents a participant and each dot a structural MRI scan. The type of scanner used is marked with a symbol. A square means that the participant was scanned on two different scanners on the same day. Age is the age at each visit. Please note that only participants above 20 years were included in the analyses, but hippocampal change was calculated from all available scans, also scans taken before the age of 20 years. The colors reflect age at each visit. Longitudinal data was used for calculation of hippocampal atrophy only.

Longitudinal data included anatomical T1-weighted scans only. 125 participants had been scanned at a Siemens 1.5 Avanto at their first timepoint (2 repeated 3D T1-weighted magnetization prepared rapid gradient echo (MPRAGE): TR/TE/TI = 2,400 ms/3.61 ms/1,000 ms, FA = 8°, acquisition matrix 192 × 192, FOV = 240 × 240 mm, 160 sagittal slices with voxel sizes 1.25 × 1.25 × 1.2 mm). These were then scanned both at the Avanto and at the Skyra at the same day to allow modelling the effect of the scanner change. At a later follow-up, 61 participants were additionally scanned on a Siemens 3T Prisma (208 sagittally oriented slices using a turbo field echo pulse sequence: TR = 2400 ms; TE = 2.22 ms; flip angle = 8°; voxel size = 0.8 × 0.8 × 0.8 mm). 24 additional participants were scanned on all three scanners (Avanto, Skyra, Prisma) to allow model scanner differences, but did not complete the fMRI task, and thus were not part of the other analyses presented in this study. We have previously shown that the correlations between hippocampal volume across the Skyra and the Avanto are high (*r* ≥ .85)^15^. In the present study, we regressed out the effect of scanner on hippocampal volume in the sample of participants scanned on more than one scanner at the same day, applied the weights to all participants undergoing scanner change between timepoints, and did all statistical analyses on the residuals. As a test of the procedure, we correlated hippocampal offset volume calculated only from Skyra scans with offset volumes including both Skyra, Avanto and Prisma scans. The correlation was *r* = .99. Hence, the approach of regressing out scanner change from the longitudinal anatomical data seems valid. A graphical presentation of the distribution of scans and scanners, including the double-scanning, is presented in Fig. 1.

### MRI preprocessing and analysis

Volumetric segmentation of the hippocampus from the T1-weighted scans was performed with FreeSurfer 7.1 (https://surfer.nmr.mgh.harvard.edu/) (Fischl et al., 2004a, 2002). DTI scans were processed with FMRIB’s Diffusion Toolbox (fsl.fmrib.ox.ac.uk/fsl/fslwiki)^31,32^. From the B0 images with reversed phase-encode blips, we estimated the susceptibility-induced off-resonance field using a method similar to what is described in (Andersson et al., 2003) as implemented in FSL (Smith et al., 2004). We then applied the estimate of the susceptibility induced off-resonance field with the eddy tool (Andersson and Sotiropoulos, 2016), which was also used to correct eddy-current induced distortions and participant head movement, align all images to the first image in the series and rotate the bvecs in accordance with the image alignments performed in the previous steps (Jenkinson et al., 2002; Leemans and Jones, 2009). After estimating the diffusion ellipsoid properties, i.e., the three eigenvalues defining the length of the longest, middle, and shortest axes of the ellipsoid, we computed mean diffusivity (MD), defined as the average of the three eigenvalues, as implemented in the FSL tool *dtifit*. The processed MD maps were co-registered to the segmented T1-weighted images to extract average MD values in the hippocampus. Specifically, for each participant, all DTI voxels for which more than 50% of the underlying anatomical voxels were labeled as hippocampus by FreeSurfer were considered representations of the hippocampus.

fMRI data was initially corrected for susceptibility distortions, motion and slice timing corrected, smoothed (5 mm FWHM) in volume space and high-pass filtered (at 0.01 Hz) using FSL (http://fsl.fmrib.ox.ac.uk/fsl/fslwiki). Next, FMRIB’s ICA-based Xnoiseifier (FIX, v1.06) (Salimi-Khorshidi et al., 2014) was used to auto-classify noise components and remove them from the fMRI data. The classifier was trained on a task-specific dataset in which task fMRI data from 36 participants had been manually classified into signal and noise components (age span in training set: 7–80; fMRI acquisition parameters identical to the current study). Motion confounds (24 parameters) were regressed out of the data as a part of the FIX routines. Transformation matrices between functional-native, structural-native and FreeSurfer average space were computed to delineate hippocampal structures and bring them to the functional-native space.

The preprocessed fMRI data was entered in a first-level general linear model (GLM) with FSFAST (https://surfer.nmr.mgh.harvard.edu/fswiki/FsFast) for each encoding and retrieval run, consisting of several conditions/regressors modeled as events with onsets and durations corresponding to the trial events during encoding and retrieval and convolved with a two-gamma canonical hemodynamic response function (HRF). At retrieval, each “old” trial (test item presented during encoding, *n* = 100) was assigned to a condition based on the participant’s response at test. Two conditions of interest were modeled both at encoding and at retrieval. (1) The source memory encoding condition consisted of items that were later correctly recognized with correct source memory (Yes response to test Questions 1 and 2 and correct response to Question 3). (2) The miss condition consisted of items presented during encoding which were not recognized during test (incorrect No response to test Question 1). In addition, several regressors were included to account for BOLD variance associated with task aspects not included in any investigated contrast. During both encoding and retrieval, an item memory condition was included that consisted of items that were correctly recognized but for which the participant had no source memory (Yes response to Question 1 and No response to test Question 2 or incorrect response to Question 3) as well as a fourth regressor that modeled trials in which the participant did not produce any response to the first question. For the retrieval runs, four additional regressors were included to model the response to the new items (i.e. correct rejections and false alarms) and to model the second and third test questions (Questions 2 and 3). Temporal autocorrelations [AR(1)] in the residuals were corrected using a prewhitening approach. For memory analyses, a contrast of interest consisting of source – miss memory conditions was computed for each participant.

### Hippocampal segmentation for fMRI

Since encoding and retrieval are known to activate anterior and posterior sections of the hippocampus to various degrees, we divided the FreeSurfer segmented hippocampus in an anterior and a posterior part. Moving anteriorly through the coronal planes of an MNI-resampled human brain, y = -21 corresponds to the appearance of the uncus of the parahippocampal gyrus. In line with recommendations for long-axis segmentation of the hippocampus in human neuroimaging^33^, we labeled hippocampal voxels at or anterior to this landmark as anterior HC while voxels posterior to the uncal apex were labeled as posterior HC. Specifically, for each participant, all functional voxels for which more than 50% of the underlying anatomical voxels were labeled as hippocampus by FreeSurfer were considered functional representations of the hippocampus. While keeping the data in native subject space, we next established hippocampal voxels’ locations relative to MNI y = -21 by calculating the inverse of the MNI-transformation parameters for a given subject’s brain and projecting the back-transformed coronal plane corresponding to MNI y = -21 to functional native space. All reported activity measures thus represent averages from hippocampal sub-regions established in native space.

### Definition of memory performance groups

Memory performance was indexed by the principal component of all the response variables from the retrieval-fMRI task (see above). We created three groups based on age-corrected memory performance. We regressed out age from the memory score, and defined participants scoring *≥* 1 SD above the age-corrected mean as high performers, 1 SD *≤* below as low performers, and the rest as normal performers. This allowed us to define high performers across the age-range, and test if the brain correlates of superior memory had commonalities across adulthood, or whether there are unique aspects to superior memory function in older age. Descriptive statistics for the groups are provided in Table 1. Our tentative division of participants +/- 1SD from the age-corrected mean fits reasonably well with a previous study using a data-driven pattern-mixture model to divide older participants in groups of maintainers (18%), decliners (13%) and average decliners (68%) based on change in performance on episodic memory tasks^10^.

In addition, a principal component was generated from two independent out-of-scanner memory tests (the California Verbal Learning Test (CVLT) learning and 30 min recall conditions (Delis, Kramer, Kaplan, & Ober, 2000) and the Rey-Osterrieth Complex Figure Test (CFT) 20 min recall condition (Meyers & Meyers, 1995) for comparison with the in-scanner memory measure. The memory factor explained 65.9% of the variance (eigenvalue 2.0), with CVLT learning (0.92) and 30 min free recall (0.92) having higher loadings than CFT (0.54). Further, a working memory factor was computed as the principal component of digit span forward, backward, and the letter memory test^34^. The working memory factor explained 65.7% of the variance (eigenvalue = 2.0), with relatively similar loadings for digit span forward (0.83), backwards (0.84) and letter memory (0.76).

### Statistical analyses

Analyses were run in R (https://www.r-project.org) using Rstudio *(*www.rstudio.com) IDE, except for an ANOVA which was run in IBM SPSS 29.0.2.0. For each participant, we calculated hippocampal slope (“atrophy”) and offset (“volume”) by fitting a linear model with hippocampal volume as dependent and time since baseline as independent variable. We then corrected hippocampal offset variables (volume) for estimated intracranial volume by running a linear regression and saving the residuals. All statistical analyses were done on the ICV-corrected hippocampal volumes. Hippocampal activity was measured separately for anterior vs. posterior hippocampus and encoding vs. retrieval. We therefore ran a repeated-measures ANOVA with phase (two levels: encoding, retrieval) × longitudinal axis (two levels: anterior, posterior) as within-subject factors and age group (two levels: older [*≥* 60 years), young/ middle-aged [20–60 years]) and memory performance group (2 levels: high, normal) as between-subject factors to disentangle the relationships to memory from the different fMRI variables. Sex and age were included as covariates. In a previous study using an overlapping sample we found only anterior hippocampal retrieval activity to be nominally related to memory performance^30^. However, that sample was much larger with a wider age-range (6.8–80.8 years) and used a different metric for memory performance, so it was necessary to test the relationship within this study.

Generalized Additive Models (GAM) using the package “mgcv”^35^ were used to derive age-functions and to test age-interactions. Group means in memory and the neuroimaging markers were compared using Tukey’s Honestly Significant Difference (Tukey HSD) method yielding adjusted p-values to control the familywise error rate (95%). Finally, we ran a GAM with memory score as continuous variable and all the hippocampal variables as predictors, with age, sex, and six variables indexing movement during the fMRI scanning as covariates.

## Results

### Descriptive group statistics

There were no significant group differences in age, but there was a higher proportion of males in the high performance group. The high performance group had a higher out-of-scanner memory score than the two other groups, demonstrating that superior memory performance was not restricted to the in-scanner memory test used for group classification. Similarly, the normal performance group showed higher out-of-scanner memory than the low performance group. The high performers also had higher working memory scores than the two other groups.

**Table 1:** Descriptive group statistics.

	Group 1: High	Group 2: Normal	Group 3: Low	1–2 *p* =	1–3 *p* =	2–3 *p* =	ANOVA *p* =
n	45	191	41				
Age	47.3	49.8	48.1	0.66	0.97	0.85	0.63
Sex (f/m)	18/27	129/62	26/15	0.001	0.06	0.87	0.003
Working mem (z)	0.37	-0.02	-0.32	0.04	0.003	0.15	0.004
Recall mem (z)	0.48	-0.02	-0.42	0.004	4.2e^−5^	0.04	6.6e^−5^

Working mem is the principal component (z-score) of out-of-scanner working memory tests, i.e. digit span forward, digit span backward and letter memory. Recall mem is the principal component (z-score) of out-of-scanner memory tests, i.e. CVLT total learning, 30 min free recall and RCFT 20 min recall. P-values for the post hoc ANOVA tests were adjusted by Tukey HSD to keep a 95% family-wise confidence level.

Behavioral results and age-correlations for the in-scanner memory task are presented in Table 2.

**Table 2:** Behavioral results.

	Mean	SD	Pearson’s *r*	*P* <
Recognition hit	73.39	12.64	-0.23	0.001
Correct rejection	89.87	9.36	-0.30	0.001
Recognition miss	22.42	11.27	0.16	0.01
False alarms	6.12	5.55	0.24	0.001
Recollection	50.75	15.36	-0.43	0.001

Behavioral results for the in-scanner memory task. A total of 100 old and 100 new items were presented, yielding a maximum score of 100. As there are occasional missing responses, the different categories do not necessarily sum to 100. The Pearson’s r column and the p-values represent the age-correlations.

### Distribution of memory scores and age-relationships

**Fig. 2:**
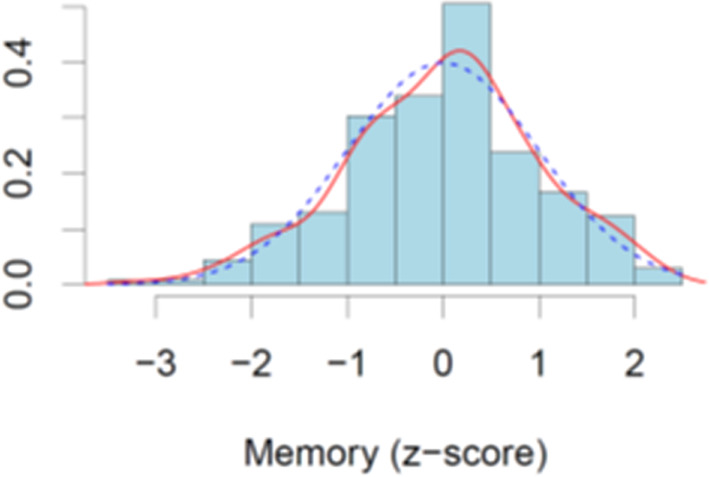
Density plot of unadjusted memory scores. The red curve shows the density distribution of the data, and the blue dotted line shown the normal distribution. Memory performance (z-scores) is on the x-axis, density on the y-axis.

The Shapiro-Wilk normality test suggested that the memory scores did not deviate from a normal distribution (W = 0.99, *p* = .34) (Fig. 2). This suggests that all participants are drawn from the same underlying distribution of memory scores, and that there are no distinct subgroups within the overall sample which exhibit different patterns of performance on the memory test.

**Fig. 3:**
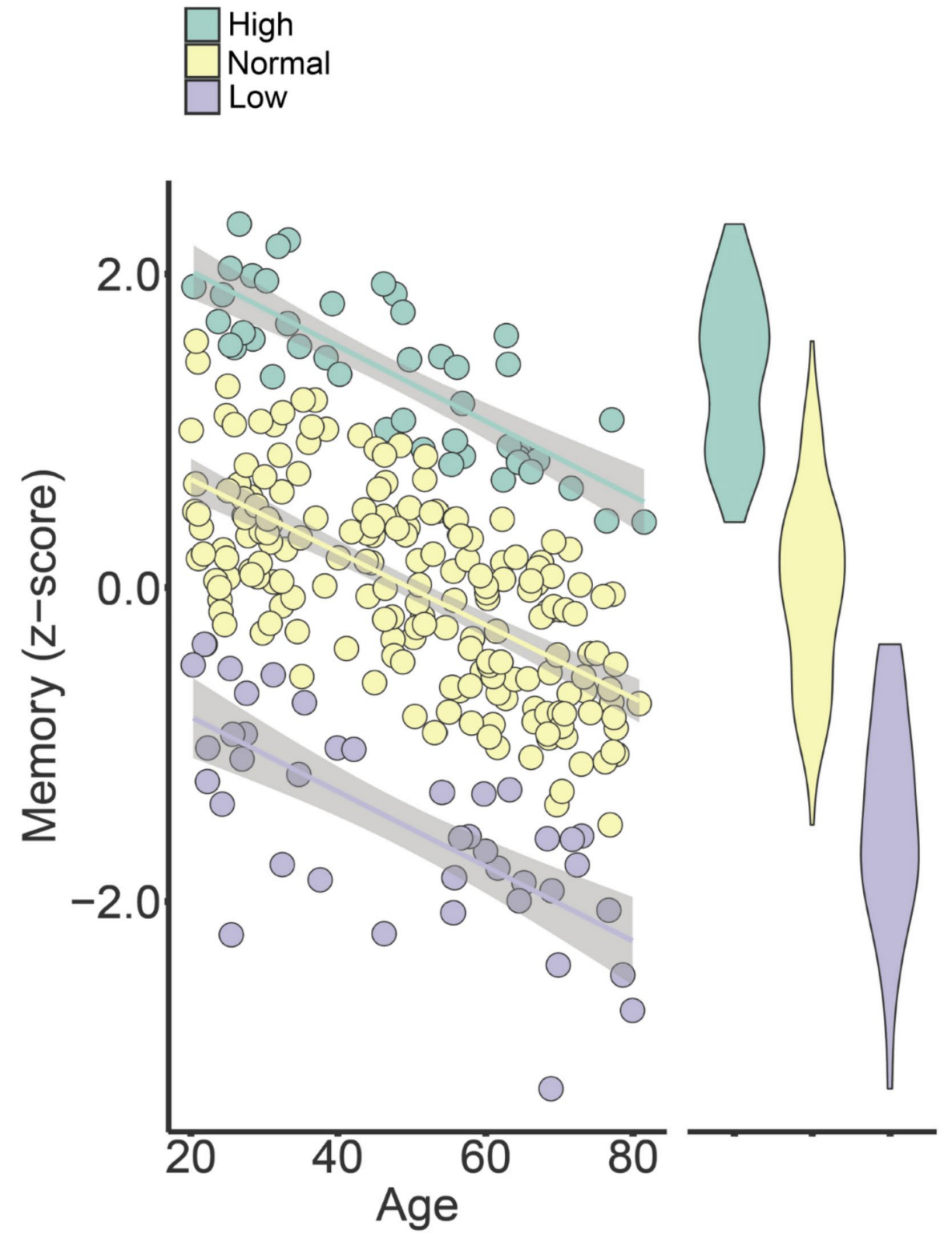
Memory across age. Scatterplots showing individual memory scores across age, with distribution of scores for each performance group illustrated by the violin plots next to the scatterplot. Color coding according to age-adjusted memory performance. Group 1 (green): high function; Group 2 (yellow): normal function; Group 3 (light purple): low function. The shaded areas around the lines depict 95% CI.

Memory was linearly and negatively related to age (Estimate = -0.023 SD change pr year, SE = 0.003, t = -7.88, *p* < 7.87e^−14^), with a correlation of *r* = − .42. The age-trajectory is shown in Fig. 3, and the individual scores are displayed with color codes according to the age-independent (right panel) performance groups.

### Age-relationships of hippocampal volume, atrophy, microstructure, and activity

Except for memory-related brain activity, all hippocampal features were significantly related to age, with steeper relationships from mid-life. Scatterplots are shown in Fig. 5 and numeric results in Table 3. There was a correlation between larger hippocampal volume and less atrophy (*r* = .21, *p* < .001). Sex was not related to any brain measure except hippocampal MD, which was significantly higher in males (t = 6.4, *p* < .6.8e^−10^).

**Table 3:** Age-relationships of hippocampus features.

Hippocampal feature	edf	F	*R* ^2^	*P*<
Volume (offset)	2.70	24.03	0.23	2e^−16^
Atrophy (slope)	3.17	40.26	0.37	2e^−16^
Microstructure (MD)	2.95	36.47	0.33	2e^−16^
Encoding-anterior	1.00	0.00	0.00	0.96
Encoding-posterior	1.00	3.20	0.01	0.07
Retrieval-anterior	1.18	2.14	0.01	0.1
Retrieval-posterior	4.10	1.84	0.03	0.1

Results of repeated GAMs with each hippocampal feature as dependent and s(Age) as independent variable. Edf: Effective degrees of freedom (expressing degree of non-linearity). P-values not adjusted for multiple comparisons.

**Fig. 4:**
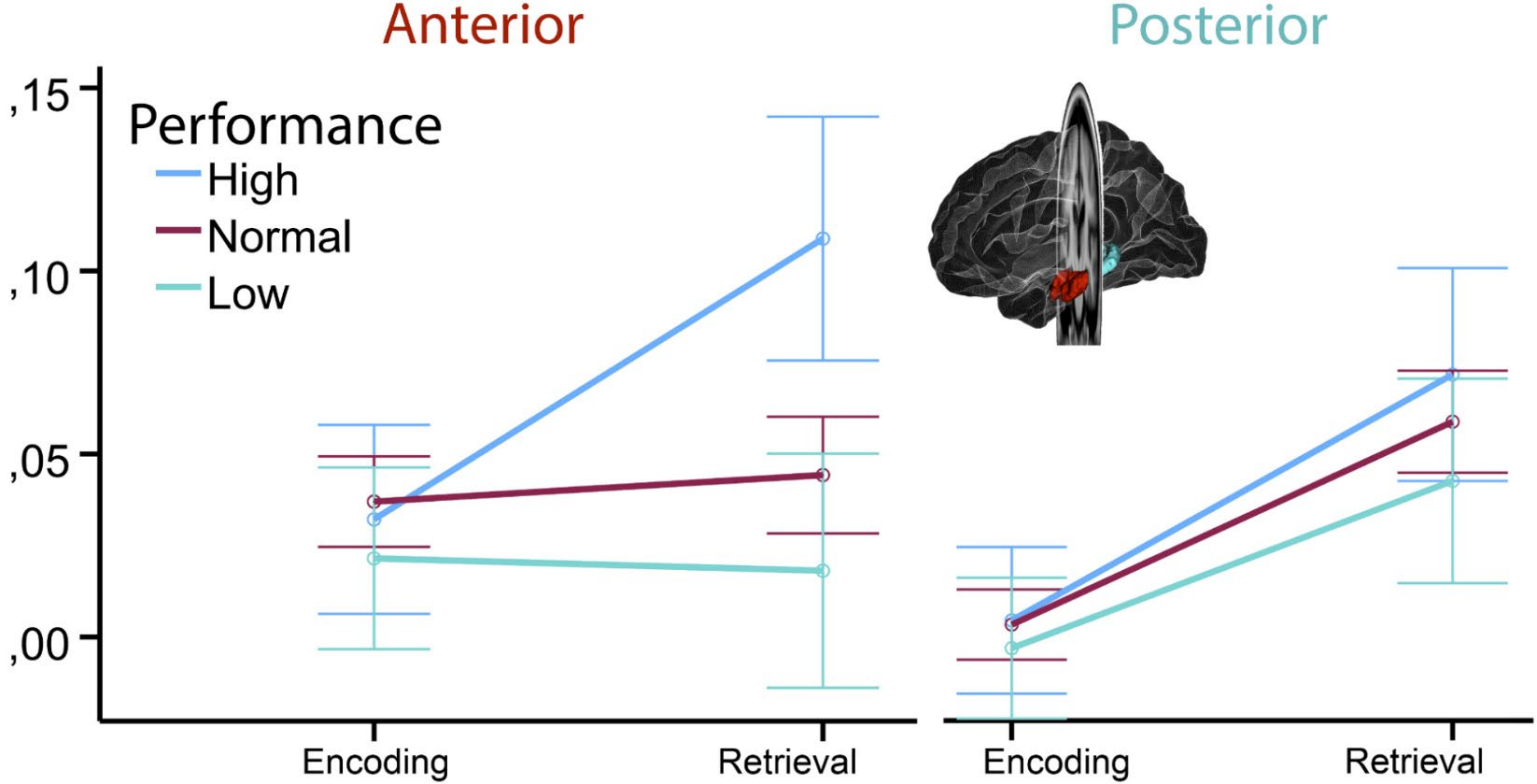
ANOVA of interactions between memory performance and memory-related fMRI activity. The figure illustrates that memory-related activity differences between participants with high vs. low memory performance tended to be larger for the retrieval phase in the anterior hippocampus. Error bars depict 95% CI.

For brain activity, the ANOVA showed a significant memory group × phase × long axis interaction (F = 4.05, *p* < .018), caused by larger effect of memory group on activity during retrieval than encoding, and this effect was larger in anterior than posterior hippocampus (see Fig. 4). There was also a main effect of memory performance group, with higher activity associated with better memory scores (F = 4.60, *p* < .011), but no memory performance × age interaction (F = 0.22, *p* = .81). Memory performance group also showed a trend towards an interaction with encoding/ retrieval (F = 2.84, *p* = .060), caused by larger group differences in retrieval than encoding activity. Post hoc tests revealed that while the high performance group showed larger anterior retrieval activity than each of the other groups (both p’s < 0.002), there were no group-differences for posterior retrieval, anterior encoding or posterior encoding (all p’s > 0.14).

### Age-independent memory performance groups

**Fig. 5:**
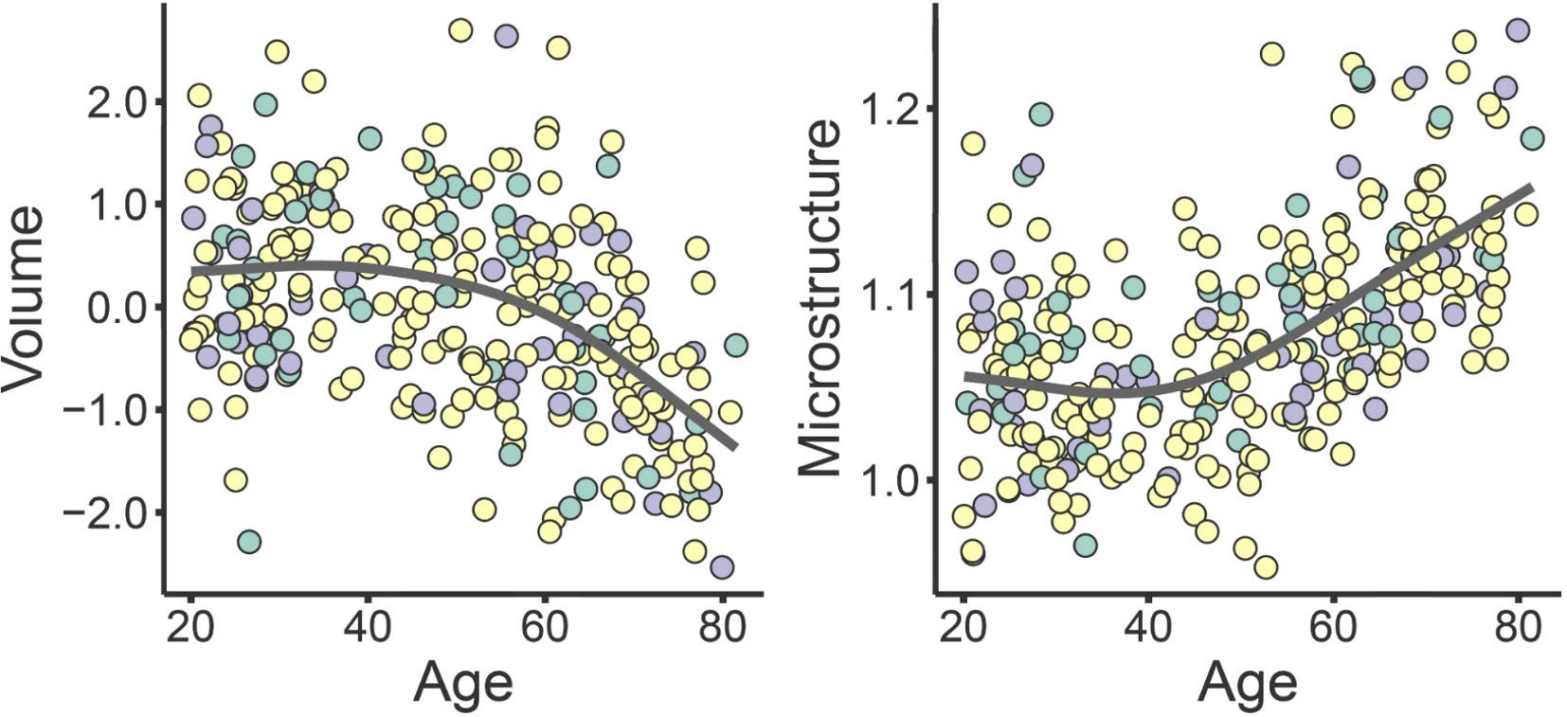
Age-relationships of hippocampal features. Scatters are color-coded by age-independent memory performance. Retrieval activity was measured in the anterior hippocampus as the contrast between successfully recollected items and forgotten items (miss). The unit for volume is z-score, for microstructure mm^2^/s ×1000, for atrophy annual change in z-score based on longitudinal MRIs. Group 1 (green): high function; Group 2 (yellow): normal function; Group 3 (dark purple): low function).

**Fig. 6:**
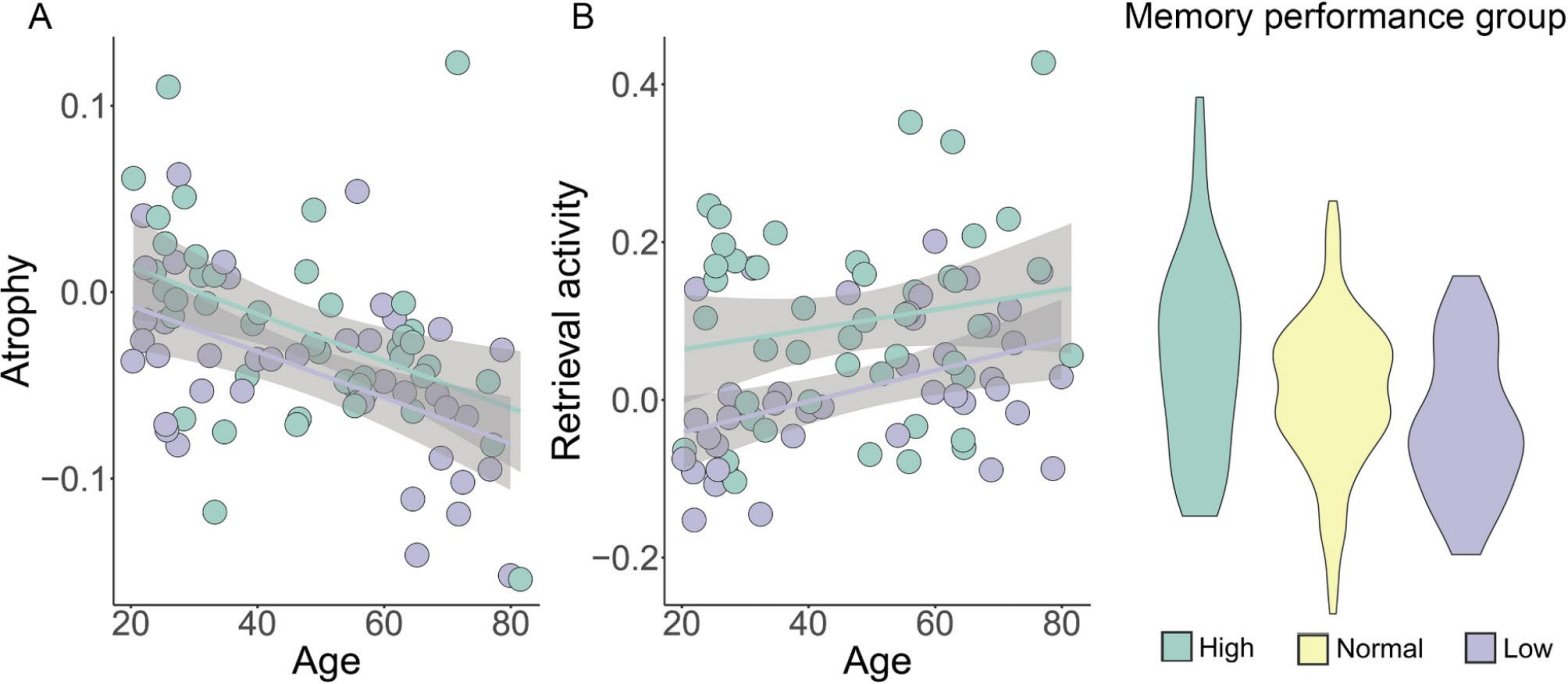
Age-trajectories for the high (green) and low (dark purple) memory performers for atrophy (left panel: A) and retrieval activity (middle panel: B). Violin plots showing the values and distribution of retrieval activity for the three age-independent memory performance groups, with the high perming group showing higher activity than the two other groups. The shaded areas around the lines depict 95% CI.

As can be seen in Fig. 3, the age-trajectories for each performance group were close to parallel. If the high memory function in aging primarily was a result of less age-related decline, we would expect a less steep age-trajectory for the high-performing group. The corresponding correlations between raw (not age-corrected) memory performance and age in each group was *r* = − .80 (high performers), *r* = − .66 (average performers), and *r* = − .72 (low performers). Main effects and age-interactions for group membership were tested by GAMs with each of the hippocampal features in turn as outcome variable (see Fig. 5 for scatterplots of age-trajectories with observations colored by performance group). There was a significant effect of memory performance group on hippocampal atrophy (estimate = 0.014, t = 2.29, *p* = .023), due to more atrophy in the low than high performing group. Performance group was also significantly related to retrieval activity (estimate = 0.06, t = 4.02, *p* = 7.34e^−5^), with both the normal and low performing group showing lower activity than the high performing group. No significant age-interactions were detected. There were no main effects or age-interactions for hippocampal volume or microstructure. All GAMs were run with both linear and tensor interactions to attempt to identify age-interactions, but no significant effects were seen. Running the analyses using memory performance as a continuous variable where age was not regressed out but rather included as a covariate gave the same main results. Memory performance was related to hippocampal atrophy (estimate = 0.005, t = 2.02, *p* = .044), with no age-interaction (*p* = .15), and to retrieval activity (estimate = 0.029, t = 4.31, *p* = 2.29e-5), also with no age-interaction (*p* = .52). For microstructure (estimate = -0.00, t = -0.11, *p* = .91) and hippocampal offset volume (estimate = 0.03, t = 0.53, *p* = .60), there were no significant main effects. The age-invariant effects of memory performance group on atrophy and retrieval activity are illustrated in Fig. 6.

Finally, the GAM with all hippocampal features as simultaneous predictors of memory score showed that hippocampal atrophy (*p* = .045) and anterior hippocampus retrieval activity (*p* = 5.38e^−5^) were unique predictors. R^2^ (adjusted) for the total model was 0.233. We also re-ran the model, including average mean diffusion in the TBSS-derived skeleton as an additional predictor, which had minor effects on the results, reducing the p-value for atrophy slightly (0.031), while retrieval activity was still highly significant (*p* = 4.46 e^−5^) and R^2^ (adjusted) = 0.237.

## Discussion

The results suggest that the same features of the hippocampus were related to superior memory across adult life, not being specific to aging. Superior memory performance showed stable associations to memory-related retrieval activity and hippocampal atrophy, and high performing older adults did not seem to represent a distinct sub-group with different brain correlates compared to high-performers in other age-groups. Results were generally in agreement whether participants were divided in age-independent memory groups or memory was analyzed as a continuous variable. Hence, analyzing population representative participants across wide age-ranges and to study normal variation using continuous variables instead of creating groups based on performance may be preferable. Implications of the results are discussed below.

### Who have superior memory in aging?

Older adults with superior memory almost certainly had superior memory also when young. We know that there is a high correlation between cognitive function early and late in life^5,36–38^, with age-invariant brain structural correlates^39^. Studies focusing on episodic memory find that many high performing older adults are not able to maintain cognitive function, and very few go from normal to very high function^12,40^. Although this cannot be directly tested in the present data of cross-sectional memory tests, it is notable that the relationship between memory and age was similar across the three age-independent performance groups. This shows that the differences between the high vs. low performing older adults are comparable to the differences between high vs. low performing younger participants. From this perspective, to have superior memory function in aging primarily requires a high starting point, while slower age-expected decline will yield some additional benefit. This is in accordance with a recent study reporting larger cortical area across life in participants with high cognitive functions, but also some evidence of less change over time^41^.

An influential model holds that maintenance of brain structure and function in late life is a primary condition for successful memory aging^4^. One study showed that although high performing older adults experienced significant cortical atrophy over 18 month, volume loss was less than in age-matched controls^14^. Here we see indications of the same phenomenon when comparing hippocampal atrophy between high and normal performing older and younger adults. When plotted across the full age-range, the atrophy-memory association turned out not to be age-specific. Thus, the results do not give strong evidence for low rate of atrophy being an important aspect of superior memory performance in aging specifically. Still, several previous studies have shown significant, although modest, correlations between rate of hippocampal atrophy and change in episodic memory function in aging^19–21^. Thus, low rate of hippocampal atrophy may be one correlate of relatively better preserved memory function, although it explains only a minor portion of the inter-individual differences in cognitively normal participants at any point in life. Memory preservation can only be addressed by use of longitudinal memory data, however. Still, a reason for the memory-atrophy relationship in the absence of a memory-volume relationship may stem from memory function being more influenced by brain change than larger hippocampal volume per se.

Further evidence for brain maintenance comes from studies reporting that preservation of functional memory networks including the hippocampus is associated with better memory function in aging^22,23^. In a previous study using an overlapping sample, Vidal-Piñeiro et al. found that frontal brain function during encoding resembling activity of younger participants was a primary characteristic of higher memory function in aging^24^. In the present study, the strongest association with high episodic memory function was found with anterior hippocampal retrieval activity. Memory-related activity was the only hippocampal feature not related to age, and the relationship with memory appeared as a stable association across the age-span. As illustrated in Fig. 3, the age-curves of high vs. low performers are parallel. This suggests that at least some of the brain basis of superior memory in aging is also associated with superior memory in young and middle age. Concurrent longitudinal data on memory function spanning several years is needed to draw stronger conclusions.

Limitations of the present work must be highlighted. First, as memory performance was based on cross-sectional data, results do not directly inform about memory change and resistance to age-decline. Second, we focused on the hippocampus as this is a key region for memory function, with established importance in aging^3^. However, many other brain regions contribute to high memory function, and it is possible that some of these would show age-variant relationships to cognition. Third, atrophy estimates often suffer from low reliability. However, long follow-up intervals substantially increase reliability in neuroimaging studies, and more so than multiple time points^42^, which likely yielded high accuracy in the atrophy estimations in the present study. Fourth, as the sample was a convenience sample, participants are above average healthy and cognitively well-functioning, and not representative of the general population. Hence, results can not automatically be generalized to other populations. Fifth, as longitudinal memory scores were not available, we cannot draw any conclusions about what causes memory *change*. Instead, we focus on whether hippocampal features relate to memory in the same way across the age-span of the sample, which can yield information about whether there are special features that are more important for memory function in aging compared to younger ages.

## Conclusion

Superior memory function in aging is related to features of hippocampal activity and atrophy in age-invariant ways. Future studies should assess changes in memory function with longitudinal designs coupled to multi-modal neuroimaging to disentangle stable factors from brain changes in older age cognitive function.

## Data Availability

Data cannot freely be shared due to data protection restrictions. Requests for access can be addressed to the corresponding author.
